# Prolonged QT interval in a man with anorexia nervosa

**DOI:** 10.1186/1755-7682-2-23

**Published:** 2009-07-31

**Authors:** María Dolores Macías-Robles, Ana María Perez-Clemente, Carmen Maciá-Bobes, María Asunción Alvarez-Rueda, Sergio Pozo-Nuevo

**Affiliations:** 1 Department of Emergencies, Hospital San Agustín, Avilés, Asturias, Spain; 2 Department of Endocrinology, Hospital San Agustín, Avilés, Asturias, Spain; 3 Department of Biochemistry, Hospital Juan Canalejo, La Coruña, Galicia, Spain

## Abstract

Anorexia nervosa is an eating disorder characterized by the avoidance of food intake, which usually leads to a weight loss. Cardiac co-morbility is common and we can find sometimes a mass loss from the left ventricle, which can be seen by echocardiography. But the commonest complications are rhythm variations, typically bradycardia with a prolonged QT interval in up to a 40% of the cases, which altogether elevates ventricular tachycardia and sudden death risk. We present the case of a male who was diagnosed with anorexia nervosa and developed asthenia, a long QT interval and also a severe both hypokalaemia and hypomagnesaemia. We intend to discuss the pathogenic paths as well as prophylactic and therapeutic measures to this potentially-lethal pathology.

## Introduction

Anorexia nervosa (AN) is an eating disorder characterized by the avoidance of food intake, which usually leads to a weight loss. AN is more common in women (10:1) and teenagers are most likely to suffer from it. Only a 5–10% of the patients are men, and it is believed that they develop eating disorders at an older age [1,2].

AN is potentially lethal and it is estimated that one third of deaths are due to cardiac events. Weight loss leads to an important muscular mass loss. There are also several electrolytic disorders due to a reduced intake and, in many cases, a parallel use of diuretic, emetic or laxative substances. All these factors are clinically reflected by weight loss, limited capacity for physical exercise and cardiac alterations. These are very common, being found in up to an 80% of the cases. The usual cardiac alterations linked to anorexia nervosa are bradycardia, hypotension, arrhytmia, prolonged QT interval (15–40%) and sudden death in up to a 10% of the cases [3-7].

We present the case of a male who was diagnosed with AN and developed asthenia with a prolonged QT interval and also severe both hypokalaemia and hypomagnesaemia. We intend to discuss the pathogenic ways as well as prophylactic and therapeutic measures to this potentially-lethal pathology.

## Case presentation

A 36-year-old man presented to the Emergency Department with a 7-day history of asthenia. He was not having any other symptom but his past medical history was significant for AN, for which he was not following any treatment. The physical examination revealed a poorly nourished male weighting 36 kg (BMI: 13,18). His temperature was 36,4°C and his blood pressure was 70/40 mmHg. His heart sounds were normal, without any murmurs, rubs or gallops.

**Figure 1:**
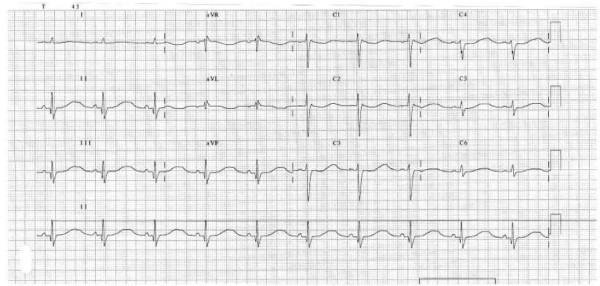
**ECG on admission showed a sinus rhythm with a heart rate of 59 beats per minute and a QT interval of 0.7 seconds**.

The laboratory tests showed: sodium 120 mmol/L (133–145), potassium 1,75 mmol/L (3,30–5,10), magnesium 0,50 mmol/L (0.65–1.05), calcium 0,99 mmol/L (1.12–1.32), total proteins 5,8 g/dL (6.6–8.7); glucose, urea, creatinine, creatine kinase, troponin T and venous gasometry were within normal range. Chest X-Ray was also normal. On admission, ECG showed a sinus rhythm with a heart rate of 59 beats per minute and a QT interval of 0.7 seconds (figure 1). On cardiac monitoring, some occasional ventricular extrasystoles were found, but there were no life-threatening arrhytmias. The patient was treated intravenously with normal saline, magnesium sulphate (2 grams in one hour) and potassium chloride (20 mmol/h during the first 6 hours and 10 mmol/h through the next 12 hours). Electrolytic disbalance and the prolonged QT interval were reverted 24 hours from admission.

## Discussion

Cardiac changes in AN patients can be divided into two groups: rhythm or structural alterations, though probably both of them are in close relation. Sinus bradycardia is one of the main findings and is one of the best known mechanisms used for energy-saving. This bradycardia comes together with a vagal hypertonia, which is reflected by an increase in pulse variability. This is potentially reversible by weight-gaining [8]. Apart from bradycardia, there are also rhythm changes in the form of atrial and ventricular extrasystoles. We can see a significant increase of the QT interval [6,9,10] in up to a 40% of the cases according to some studies. This, altogether with sinus bradycardia, can trigger polymorphic ventricular tachycardias, thus increasing the risk of sudden death [6].

Structural changes have been identified by echocardiography. It has been demonstrated that weight loss comes together with a mass loss in the left ventricle [6] which, altogether with a low cardiac output, can be responsible for the asthenia. Despite this, cardiac failure with clinical consequences is rarely found [9]. After weight-gaining, at least during the initial phase of AN, these changes are potentially reversible, though the possibility of a complete regression is still unknown [8,9]. Prolonged QT interval represents local differences in myocardial excitability, which sets the ground for arrhythmia, thus increasing the risk of ventricular tachycardia and sudden death.

The aetiology of prolonged QT interval in AN is still in controversy [5,6], though it can be explained by several mechanisms. First of all, ventricular mass loss leads to a non-homogeneous repolarization. Also, it could be the consequence of changes in ion channels that set an increase of the action potential [6]. To conclude, ionic alterations in these patients, specially hypokalaemia but also hypomagnesaemia and hypocalcaemia, are well-known causes for a prolonged QT interval [9,11].

In clinical practice, prevention of arrhythmias and sudden death in AN patients must be based in a strict control of risk factors. Thus, every AN patient in the Emergencies Department should have an ECG done, and blood levels for sodium, potassium, calcium and magnesium. Also, hospital admission should be based not only in denourishment or a bad response to psychotherapy, but also in the presence of any alarm sign such as syncope, bradycardia, prolonged QT or ionic alterations. When an important hypokalaemia or hypomagnesaemia are seen, treatment is an emergency and should be always done under electrocardiographic monitorization. Magnesium levels should be corrected simultaneously with potassium infusion, for hypomagnesaemia can also lead to refractory hypokalaemia and hypocalcaemia.

According to all this, it is necessary to remind that AN is a disease that requires a careful clinical evaluation, and that we should be alert when identifying new cases. No wonder about patients presenting with denourishment, syncope, ECG changes or ionic alterations at the Emergencies Department. But generalist physicians in Primary Health Care should keep an eye on inespecific symptoms such as asthenia. Given the low rate comparing to women, AN in men is often an overlooked issue during doctors' training. On the contrary, we see that it should be considered in the differential diagnosis of young men complaining of sudden onset tiredness, as we can prevent serious complications by detecting and treating it.

## Competing interests

The authors declare that they have no competing interests.

## Authors' contributions

MDMR and AMPC selected the case and contributed to redaction and bibliographic research. CMB and MAAR contributed to redaction and bibliographic research. SPN did the manuscript review and the English translation. All authors read and approved the final manuscript.
